# A survey of the mycobiota associated with larvae of the black soldier fly (*Hermetia illucens*) reared for feed production

**DOI:** 10.1371/journal.pone.0182533

**Published:** 2017-08-03

**Authors:** Ilaria Varotto Boccazzi, Matteo Ottoboni, Elena Martin, Francesco Comandatore, Lisa Vallone, Thomas Spranghers, Mia Eeckhout, Valeria Mereghetti, Luciano Pinotti, Sara Epis

**Affiliations:** 1 Department of Biosciences, University of Milan, Milan, Italy; 2 Department of Health, Animal Science and Food Safety, University of Milan, Milan, Italy; 3 Pediatric Clinical Research Center Romeo and Enrica Invernizzi, University of Milan, Milan, Italy; 4 Department of Crop Protection, Ghent University, Gent, Belgium; 5 Department of Applied Biosciences, Ghent University, Gent, Belgium; 6 Department of Agricultural and Environmental Sciences-Production, Landscape, Agroenergy, University of Milan, Milan, Italy; Universita degli Studi di Camerino, ITALY

## Abstract

Feed security, feed quality and issues surrounding the safety of raw materials are always of interest to all livestock farmers, feed manufacturers and competent authorities. These concerns are even more important when alternative feed ingredients, new product developments and innovative feeding trends, like insect-meals, are considered. The black soldier fly (*Hermetia illucens*) is considered a good candidate to be used as feed ingredient for aquaculture and other farm animals, mainly as an alternative protein source. Data on transfer of contaminants from different substrates to the insects, as well as the possible occurrence of toxin-producing fungi in the gut of non-processed insects are very limited. Accordingly, we investigated the impact of the substrate/diet on the intestinal mycobiota of *H*. *illucens* larvae using culture-dependent approaches (microbiological analyses, molecular identification through the typing of isolates and the sequencing of the 26S rRNA D1/D2 domain) and amplicon-based next-generation sequencing (454 pyrosequencing). We fed five groups of *H*. *illucens* larvae at the third growing stage on two substrates: chicken feed and/or vegetable waste, provided at different timings. The obtained results indicated that *Pichia* was the most abundant genus associated with the larvae fed on vegetable waste, whereas *Trichosporon*, *Rhodotorula* and *Geotrichum* were the most abundant genera in the larvae fed on chicken feed only. Differences in the fungal communities were highlighted, suggesting that the type of substrate selects diverse yeast and mold genera, in particular vegetable waste is associated with a greater diversity of fungal species compared to chicken feed only. A further confirmation of the significant influence of diet on the mycobiota is the fact that no operational taxonomic unit common to all groups of larvae was detected. Finally, the killer phenotype of isolated yeasts was tested, showing the inhibitory activity of just one species against sensitive strains, out of the 11 tested species.

## Introduction

*Hermetia illucens* (Diptera; Stratiomyidae) is a fly, commonly called black soldier fly (BSF), which is native of the tropical and warm temperate zones of America, but it is now widespread in many regions of the world [1]. It is regarded as a good candidate for the conversion of food waste into valuable biomass; in fact BSF larvae can grow on different organic waste and manure, and convert them into insect larval biomass that can be used for different purposes [2]. In addition, these insects have shown to be able to produce bacteriostatic, bactericidal and fungicidal compounds—useful to reduce possible contaminating microorganisms—which make these insects extremely resistant to different environmental conditions [3,4]. BSF larvae have also been detected for their potential use in the feed sector: insect-meal could replace in part fish-meal and soy bean-meal as a protein source in feed production and formulation [5].

Insects as feed ingredients have great potential for different reasons: (i) nutrients content, they are rich in proteins, fat (and in turn energy), vitamins and minerals; (ii) higher feed conversion efficiency compared to livestock; (iii) low space requirement; (iv) great acceptance from poultry and fish, whose diet in nature is partly represented by insects; (v) they are mostly omnivorous and can grow on different substrates [6]. While the nutritional properties of edible insects have been documented in several studies [7,8], to date their safety is less investigated [9]. The consumption of insects could represent a microbial, parasitical and chemical hazard; insects are indeed associated with different microorganisms (bacteria, fungi and virus) or could contain toxic and repellent substances, which are part of their defense repertoire [6,10].

Recently, several studies have focused on the impact of ecological determinants like diet, host/rearing environments and life stage on the insect gut microbiota [11,12]. Concerning BSF larvae, while the effect of different substrates on the intestinal bacterial community has already been investigated [4], fungal biodiversity has not yet been studied. Fungal community (mycobiota) in the gut of insects can play important roles in detoxification of metabolites and supply of enzymes, essential amino acids, vitamins and sterols in the host's diet [13]; however, fungi are also able to produce toxins, which can enter the trophic network and represent a risk for animals and humans [14].

Accordingly, the aims of the present study were: (i) to study the intestinal mycobiota of BSF larvae raised for different times on two diverse substrates, chicken feed and/or vegetable waste; (ii) to characterize the fungal community also in terms of anti-microbial activity.

## Materials and methods

### Experimental design

BSF larvae used in this study were provided by a stock colony established at the Department of Crop Protection, Faculty of Bioscience Engineering of Ghent University, Gent (Belgium).

**Fig 1:**
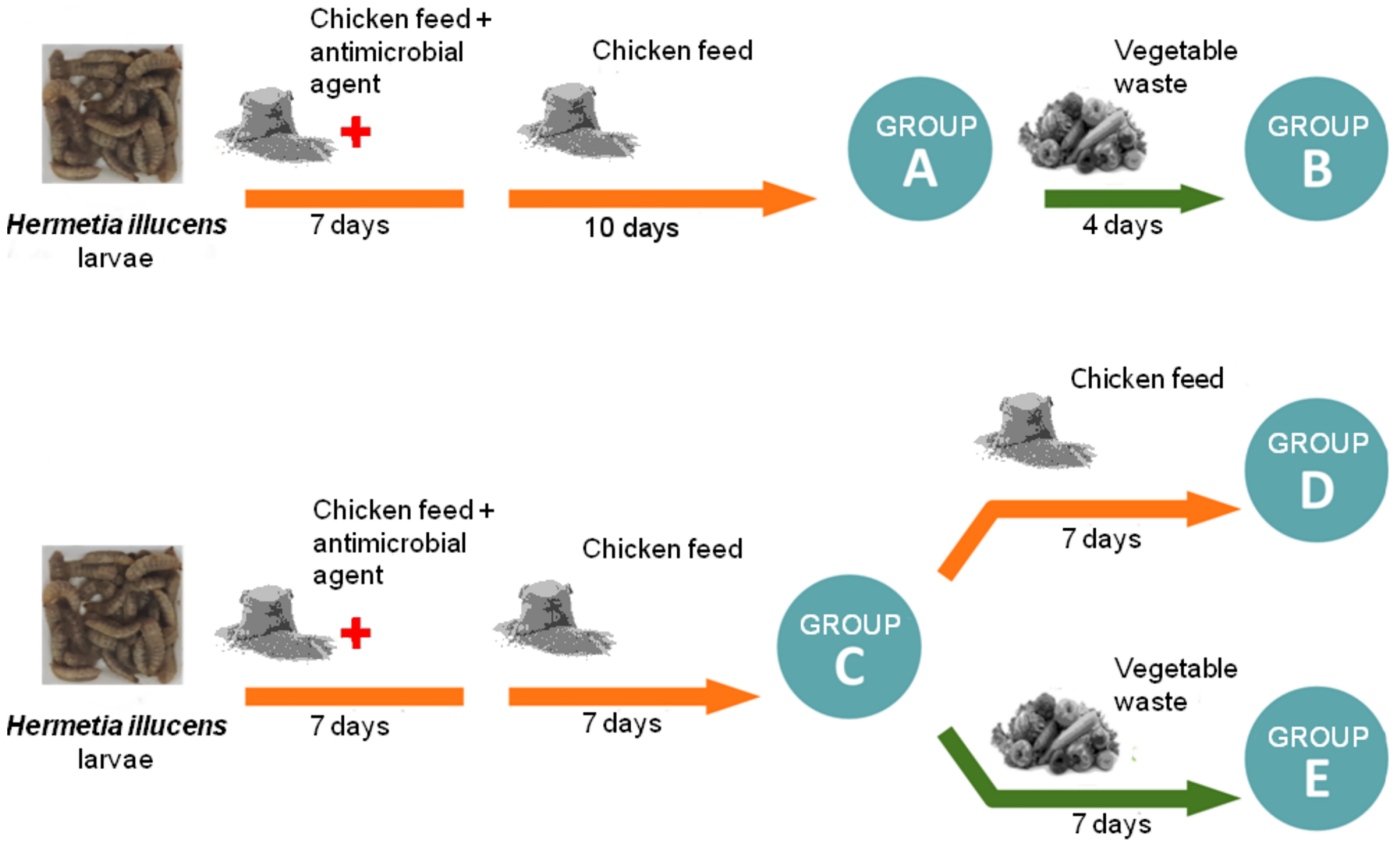
Experimental design. Groups of *Hermetia illucens* larvae (A, B, C, D, E) and the different feeding conditions.

In order to study the impact of different diets and exposure times on the intestinal fungal community of BSF larvae, the following five different feeding conditions were used: chicken feed for 17 days (group A), chicken feed for 17 days and vegetable waste for the following four days (group B), chicken feed for 14 days (group C), chicken feed for 21 days (group D), chicken feed for 14 days and vegetable waste for the next seven days (group E). The experimental design and groups are reported in Fig 1.

Larvae of *H*. *illucens* (about 1000 larvae, see below) were grown on two different substrates, namely: a chicken feed diet, which has been selected as a reference substrate; a vegetable waste substrate, used as low value organic waste material. This is in line with other recent studies [15] in which chicken feed was used as reference complete (providing all the nutrients required) feed for growing insects. Moreover, as commercial feed for poultry, it meets all requirements in terms of safety (i.e. residue, mycotoxins, etc.). On the other hand, vegetable waste substrate should be considered one of the best substrate candidate in terms of sustainability. In this respect, insects can potentially be used to upgrade low value organic waste materials [7]. However, as reported by others [15], vegetable waste substrate/diet does not guarantee a fast insect growth. In light of these considerations, different chicken feed and vegetable waste combinations, at different exposure times, have been tested. The chicken feed was a layer hen feed containing 155 g/kg crude protein on ‘as is’ basis (Legkorrel TOTAL 77; Aveve Veevoeding, Merksem, Belgium). Water was added to the chicken feed (70 ml/100 g of substrate) in order to guarantee an optimal moisture content for the growth of the larvae. Vegetable waste was collected from the student restaurant of the Ghent University and contained heat-treated vegetables (mashed potatoes, steam or oven cooked carrots and tomatoes); according to the (EC) Regulation N° 1069/09 [16] animal products were not included in these substrates. In addition, methylparaben, a broad-spectrum antimicrobial agent, was blended with all substrates (4 g per 1000 g of feed) on day 0 of the experiment and administered to all larvae for the first seven days.

About 1000 BSF larvae aged six to eight days were added to 600 g of each substrate and placed into a plastic bucket under controlled conditions of temperature (27°C±1°C) and humidity (65±5%). Eight larvae from each group were collected randomly and, after anaesthetization at -20°C, were surface-sterilized in 100% ethanol and then washed twice in sterile 1X phosphate-buffered saline (PBS). The gut of each larva was dissected, washed in sterile 1X PBS and finally transferred to 300μl of the enrichment medium GLY (25g/L glycerol, 10g/L yeast extract).

### Microbiological analyses

In order to isolate yeasts and molds associated with *H*. *illucens*, two different media were employed, respectively: (a) YPD agar medium (20g/L peptone, 10g/L yeast extract, 20g/L glucose, 20g/L agar) and (b) Malt 2% (M_2_) agar medium (20g/L malt, 3g/L yeast extract, 15g/L agar), both supplemented with chloramphenicol (100mg/L) to avoid bacterial growth. Each gut was individually homogenized with a sterile pestle in GLY medium and appropriate 10-fold dilutions of the homogenized samples were produced in sterile 0.9% NaCl water solution. One hundred μl of each dilution were spread directly on YPD and M_2_ agar plates, which were incubated at 26°C for 48 h and at 28°C for five days, respectively. Growing yeasts were then selected, collected based on morphology [17] and re-plated twice on the same agar medium. Pure cultures were picked up and then inoculated in YM broth (5g/L peptone, 3g/L yeast extract, 3g/L malt extract, 10g/L glucose). After a incubation at 26°C for 12 h, the cultures were stored in 20% glycerol (Sigma-Aldrich Corp., St. Luis, USA) at -80°C. Indeed, the filamentous fungi were identified using traditional mycological methods, handbooks and identification keys based on colony characteristics and microscopic morphology [18,19].

### DNA extraction from yeasts

An aliquot of each liquid culture medium (1500μl) was transferred to a tube and centrifuged at 5000g for 10 min. The precipitated material was resuspended in 600μl of lysis solution containing 1M sorbitol, 0.1M EDTA, 14mM β-mercaptoethanol and 200U lyticase (Sigma-Aldrich Corp., St. Luis, USA) and then incubated at 30°C for 45 min. After a centrifugation at 300g for 10 min, the obtained spheroplasts were resuspended in 70μl of PBS plus 5μl of proteinase K (20mg/ml). The samples were incubated first at 56°C for 30 min and then at 95°C for 5 min. After a centrifugation at 6000g for 1 min, supernatant DNA was transferred to a new tube for the molecular analyses.

The concentration and purity of the extracted DNA were determined by a NanoDrop 2000 spectrophotometer (Thermo Fisher Scientific, Waltham, MA, USA).

### PCR amplification and ITS-RFLP analysis

Amplification of the 5.8S-ITS region was achieved with the primers ITS1F (5′-CTTGGTCATTTAGAGGAAGTAA-3′) and ITS4 (5′-TCCTCCGCTTATTGATATGC-3′) [20]. PCR was performed in 30μl of reaction mixture containing 1X Green GoTaq Reaction Buffer, 0.2mM dNTPs, 0.5μM of each primer, 1.25U GoTaq G2 (Promega Corp., Madison, WI, U.S.A.) and 50ng of template DNA extracted as above, under the following cycling conditions: 94°C for 7 min, 35 cycles at 94°C for 45 sec, 55°C for 45 sec and 72°C for 1 min with a final extension at 72°C for 10 min. Aliquots of 5μl of each amplified product were electrophoretically separated on 1.5% agarose gels in 1X TAE buffer at 90V constant voltage for 30 min.

PCR products of the 5.8S-ITS region were digested with the restriction endonucleases *Hae*III (10U/μl), *Dpn*I (10U/μl) and *Dde*I (10U/μl) (Promega Corp., Madison, WI, U.S.A.), in 20μl of reaction volume according to manufacturer instructions and conditions. Restriction fragments (ITS-5.8 RFLP’s) were separated on 2% agarose gel in 0.5X TBE buffer for 2 h. Band sizes were estimated by comparison against Bench Top 100bp DNA ladder (Promega Corp., Madison, WI, U.S.A.). Amplification of 26S rRNA D1/D2 domain of three isolates of each restriction profile was carried out using the following primer pairs: NL-1 (5′-GCATATCAATAAGCGGAGGAAAAG-3′) and NL-4 (5'-GGTCCGTGTTTCAAGACGG-3') [21]. PCR was performed in 25μl of reaction mixture containing 1X Green GoTaq Reaction Buffer, 0.2mM dNTPs, 0.5μM of each primer, 1.25U GoTaq G2 (Promega Corp., Madison, WI, U.S.A.) and 50ng of template DNA, under the following cycling conditions: 94°C for 7 min, 35 cycles at 94°C for 45 sec, 57°C for 45 sec, 72°C for 45 sec and a final step at 72°C for 10 min. Two amplicons of each profile, obtained from 26S rRNA gene, were sequenced; the resulting sequences were subjected to Blast analysis (http://www.ncbi.nlm.nih.gov/blast) and compared with sequences available in GenBank (nucleotide collection nr/nt; http://www.ncbi.nlm.nih.gov/genbank/).

### Killer activity assay

Three strains, randomly selected, of each isolated yeast species, were used for inhibition assays to test their killer phenotype, using the following sensitive reference strains (chosen according to published papers [22–28]): NEQAS 8706 (*Candida glabrata*), NEQAS 6208 (*Candida lusitaniae*), NCYC 1006 (*Saccharomyces cerevisiae*) and *Wa*UM3 [*Wickerhamomyces anomalus* (a strain not producing toxins)]. The killer strain *Wa*F17.12 (*W*. *anomalus*) was used as killer toxin positive control [29].

The killing assays were performed following the method described in Polonelli *et al*. [30] and reported with minor modifications in Martin *et al*. [23]. Briefly, each sensitive strain was resuspended in sterile 1X PBS until a final OD_530_ value of 0.15–0.18 and then plated on methylene blue agar medium (MBA): (20g/L peptone, 20g/L glucose, 10g/L yeast extract, 30g/L agar, 0.003% methylene blue, buffered at pH 4.5 with 0.2M citric acid and 1M Na_2_HPO4). Three strains of each yeast species, were spotted in duplicate on the surface of previously prepared MBA medium and the plates were incubated at 26°C for 48 h. Presence of an inhibition zone around the spot of supposed killer yeast was suggestive of an antimicrobial activity.

### DNA extraction from insect guts and Next Generation Sequencing 454 technology

The variability of the mycobiota of BSF larvae was also evaluated by Next Generation Sequencing 454 technology, using the fungal ribosomal ITS region as a target. The analysis was performed on four guts of BSF larvae for each group (A, B, C, D, E), dissected as previously described. After the dissection, each gut was carefully washed in sterile 1X PBS and homogenized with a sterile pestle. Total DNA was extracted using the enzyme lyticase (Sigma-Aldrich Corp., St. Luis, USA) and the DNeasy Blood and Tissue Kit (Qiagen, Hilden, Germany) according to manufacturer instructions and eluted in 25μl of AE buffer. The concentration and purity of the extracted DNA were determined by a NanoDrop 2000 spectrophotometer (Thermo Fisher Scientific, Waltham, MA, USA).

A commercial service performed the 454 pyrosequencing by Roche 454 GS FLX Titanium (MR DNA, Shallowater, TX-USA). The 5.8S-ITS rRNA gene sequences obtained by the 454 pyrosequencing assays were deposited in the European Nucleotide Archive (study accession n°: PRJEB20375; sample accession n°: ERS1660771; experiment accession n°: ERX1981522; run accession n°: ERR1923795). Using the QIIME platform [31], the obtained raw 5.8S-ITS rRNA gene sequences were trimmed to remove adaptors, low quality base calls (<30 Phred score) and short-sized sequences (< 200 bp). The high-quality 454 sequences were clustered into operational taxonomic units (OTUs) using *Uclust* [32] with a 97% sequence-identity threshold. A representative sequence of each OTU was selected to build the overall OTU table. These OTUs were taxonomically assigned by RDP Classifier PyNast (https://rdp.cme.msu.edu/) [33] using, as reference, the Warcup ITS fungal database [34].

### Diversity and statistical analyses

The resulting raw OTUs table was filtered in order to remove OTUs with less than five sequences and occurring in a single sample. The filtered OTU table was used as input for the analyses carried out with the R package vegan [35]. Shannon H index [36], Pielou's evenness [37] and the estimated species richness index Chao 1 [38,39] were computed.

The OTU table was transformed into a presence-absence matrix in order to be processed for the following analyses. The similarity between the microbial communities associated with the five groups of specimens was analyzed through a hierarchical cluster analysis, using the function hclust in R stats-package (R Project 3.0.2; http://cran.r-project.org/). The dissimilarity matrix, used as input for the hierarchical cluster analysis, was estimated by vegdist using the Bray and Curtis dissimilarity index. As described in Montagna *et al*. [40], in order to test the significant dissimilarity between the fungal communities associated with the five groups of insects, the dissimilarity matrix was subjected to a nonparametric one-way analysis of similarity (ANOSIM [41]). In addition, the fungal communities associated with the BSF samples were ordinated, according to their similarity in OTU composition (presence/absence OTU table used as input), by the distance-based non-metric multi-dimensional scaling method (NMDS [42]) using the Bray-Curtis distance [43]. The correlation between mycobiota composition and the diet was investigated by fitting the previous NMDS ordination scores with the *envfit* function in vegan (9999 permutations). In order to investigate the common OTUs present in the five groups tested in this study, an analysis of commonality was performed and visualized through a Venn diagram using the *gplots* package in R. In the Venn diagram, the presence was assigned when the OTU was reported for at least two specimens of the group fed on the same substrate.

## Results

### Identification of fungal species

A total of 63 yeast isolates with different morphologies was randomly picked up from YPD plates. PCR products of the 5.8S-ITS region from all isolates were generated and subsequently digested with the three restriction endonucleases *Hae*III, *Dpn*I and *Dde*I. RFLP analysis was used to discriminate yeast species by comparing the restriction pattern of each isolated strain analyzed with each enzyme; in this way, 11 different profiles were obtained. In order to determine the identity of each profile recognized by restriction analysis of the ITS region, the 26S rRNA D1/D2 domain was sequenced. The profiles were identified as: *Trichosporon jirovecii* (I), *Rhodotorula mucilaginosa* (II), *Trichosporon asahii* (III), *Pichia fermentans* (IV), *Kazachstania servazzii* (V*)*, *Kluyveromyces hubeiensis* (VI), *Pichia kluyveri* (VII), *Pichia kudriavzevii* (VIII), *Candida tropicalis* (IX), *Meyerozyma guilliermondii* (X) and *Geotrichum candidum* (XI) as reported in Table 1.

**Table 1:** RFLP analysis of ITS region of yeast isolates.

Restriction profile	Number of isolates	Groups of larvae	Species	Restriction fragments (bp)		
				*Hae*III	*Dpn*I	*Dde*I
I	3	A	*Trichosporon jirovecii*	600	500	1500+600+500
II	2	A	*Rhodotorula mucilaginosa*	460+220	800+700+600	1000+700+500+400+170+160
III	8	A-B-C-D	*Trichosporon asahii*	550+90	537	1500+1400+650+500
IV	12	B-E	*Pichia fermentans*	375+100	1900+500	700+400+300+170+100
V	3	B-E	*Kazachstania servazzii*	610+320+220	900	1500+1400+700+600+200
VI	6	B	*Kluyveromyces hubeiensis*	680+600+400+200+160	1600+800	1500+1400+700+600+160
VII	17	B-E	*Pichia kluyveri*	500+400+100	500	700+400+300+160+ 100
VIII	2	B	*Pichia kudriavzevii*	430+100+75	546	1500+600
IX	2	C	*Candida tropicalis*	482+80	562	442+115
X	4	C-D-E	*Meyerozyma guilliermondii*	390+152+61	624	430+194
XI	4	C-D	*Geotrichum candidum*	409	409	356+53

In this table are reported yeast species associated with 11 restriction profiles obtained after digestion of ITS region with the three endonucleases, lengths (in bp) of the fragments, number of isolates and corresponding groups of larvae.

All sequences showed an identity of about 99–100% with sequences available in GenBank database. A total of 22 species-specific 26S rRNA region sequences was submitted to GenBank (Table 2).

**Table 2:** Identification of yeast isolates by the 26S rRNA D1/D2 domain sequence analysis.

Yeast species	GenBank acc. no.	26S D1/D2 sequence comparison	
	D1/D2 26S	Ident (%)	Species (GenBank acc. no.)
*Trichosporon jirovecii*	LT839043	99	*Trichosporon jirovecii* (KM821099.1)
*Trichosporon jirovecii*	LT839044	99	*Trichosporon jirovecii* (KM821099.1)
*Rhodotorula mucilaginosa*	LT839045	99	*Rhodotorula mucilaginosa* (KP990660.1)
*Rhodotorula mucilaginosa*	LT839046	99	*Rhodotorula mucilaginosa* (KP990660.1)
*Trichosporon asahii*	LT839047	99	*Trichosporon asahii* (KU316752.1)
*Trichosporon asahii*	LT839048	99	*Trichosporon asahii* (KU316752.1)
*Pichia fermentans*	LT839035	99	*Pichia fermentans* (KM655842.1)
*Pichia fermentans*	LT839036	99	*Pichia fermentans* (KM655842.1)
*Kazachstania servazzii*	LT839037	99	*Kazachstania servazzii* (HM146913.1)
*Kazachstania servazzii*	LT839038	99	*Kazachstania servazzii* (HM146913.1)
*Kluyveromyces hubeiensis*	LT839039	99	*Kluyveromyces hubeiensis* (KC494714.1)
*Kluyveromyces hubeiensis*	LT839040	99	*Kluyveromyces hubeiensis* (KC494714.1)
*Pichia kluyveri*	LT839041	100	*Pichia kluyveri* (KP171598.1)
*Pichia kluyveri*	LT839042	100	*Pichia kluyveri* (KP171598.1)
*Pichia kudriavzevii*	LT839049	100	*Pichia kudriavzevii* (KP324972.1)
*Pichia kudriavzevii*	LT839050	100	*Pichia kudriavzevii* (KP324972.1)
*Candida tropicalis*	LT839051	100	*Candida tropicalis* (KP990661.1)
*Candida tropicalis*	LT839052	100	*Candida tropicalis* (KP990661.1)
*Meyerozyma guilliermondii*	LT839053	100	*Meyerozyma guilliermondii* (KU687383.1)
*Meyerozyma guilliermondii*	LT839054	100	*Meyerozyma guilliermondii* (KU687383.1)
*Geotrichum candidum*	LT839055	100	*Geotrichum candidum* (LC125946.1)
*Geotrichum candidum*	LT839056	100	*Geotrichum candidum* (LC125946.1)

The 16 mold isolates were morphologically identified as *Geotrichum candidum*, which had been already isolated on YPD plates; in fact, this fungus can display a wide phenotypic variability, from the yeast-like form to the mould-like form, according to environmental conditions. In addition, it is not considered a foodborne pathogen or mycotoxin producer [44].

### Killing assay

As reported on published papers, seven yeast species out of 11 are reported to have an antimicrobial activity against different yeast and/or bacteria strains: *T*. *jirovecii* [24], *R*. *mucilaginosa* [25], *T*. *asahii* [26], *P*. *fermentans* [26], *P*. *kluyveri* [27], *P*. *kudriavzevii* [28] and *M*. *guilliermondii* [45]. To establish whether the strains isolated in this study display a killer phenotype, susceptible yeast strains were used for specific killer phenotype assays. Susceptible strains, *C*. *glabrata* (NEQAS 8706) and *C*. *lusitaniae* (NEQAS 6208), were used to test the antimicrobial activity of *P*. *fermentans*, *T*. *asahii* and *R*. *mucilaginosa;* in fact, as reported in [25] and [26] these isolated yeast species have been tested against yeasts of the genera *Candida*.

**Fig 2:**
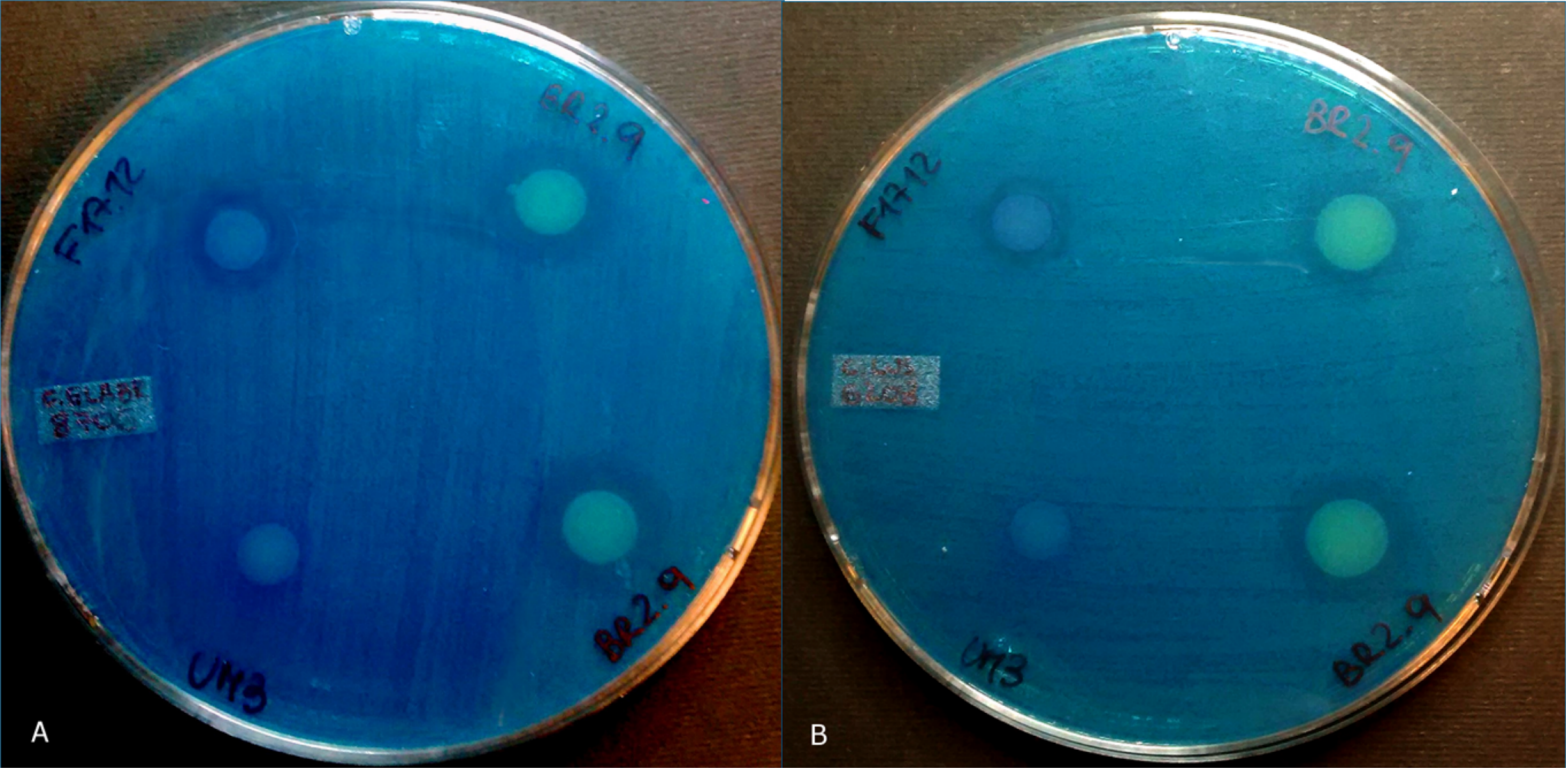
Antifungal activity of *Trichosporon asahii*. Evidence of growth inhibition of the strain *T*. *asahii* (BR2.9) (on the top and bottom right of each plate) against the susceptible strains (A) *Candida glabrata* (NEQAS 8706) and (B) *Candida lusitaniae* (NEQAS 6208). Inhibition halo of the KT-producing positive control *Wa*F17.12 (*Wickerhamomyces anomalus*) is shown on the top left of each plate. No growth inhibition was produced by *Wa*UM3 (a strain not producing toxins) (on the bottom left of each plate).

The susceptible yeasts *S*. *cerevisiae* (NCYC 1006) and *W*. *anomalus* (*Wa*UM3) were used to test *T*. *jirovecii*, *P*. *kluyveri*, *P*. *kudriavzevii* and *M*. *guilliermondii;* the first sensitive yeast was chosen according to [24,27,28] whereas the second one is a strain already used as susceptible yeast in Polonelli *et al*. [22] and Martin *et al*. [23]. After the killing assay, only three yeast strains of the species *T*. *asahii* displayed an inhibitory activity against susceptible strains. The transparent halo was similar to that generated by the positive control (*Wa*F17.12), while for the negative control (*Wa*UM3) no inhibition halo was detectable (Fig 2).

### ITS profiling, community structure and composition

A total of 90,023 raw reads of the 5.8S-ITS rRNA region was obtained. After removal of low quality reads (<200 bp), adaptors and low quality base calls (<30 Phred score), a total of 33,685 high-quality reads was analyzed. The clustering of the reads at 3% of divergence revealed a total of 303 OTUs. During the sequencing some samples of the group D and one sample of the group A did not overcome the quality control due to the weak number of sequences.

**Fig 3:**
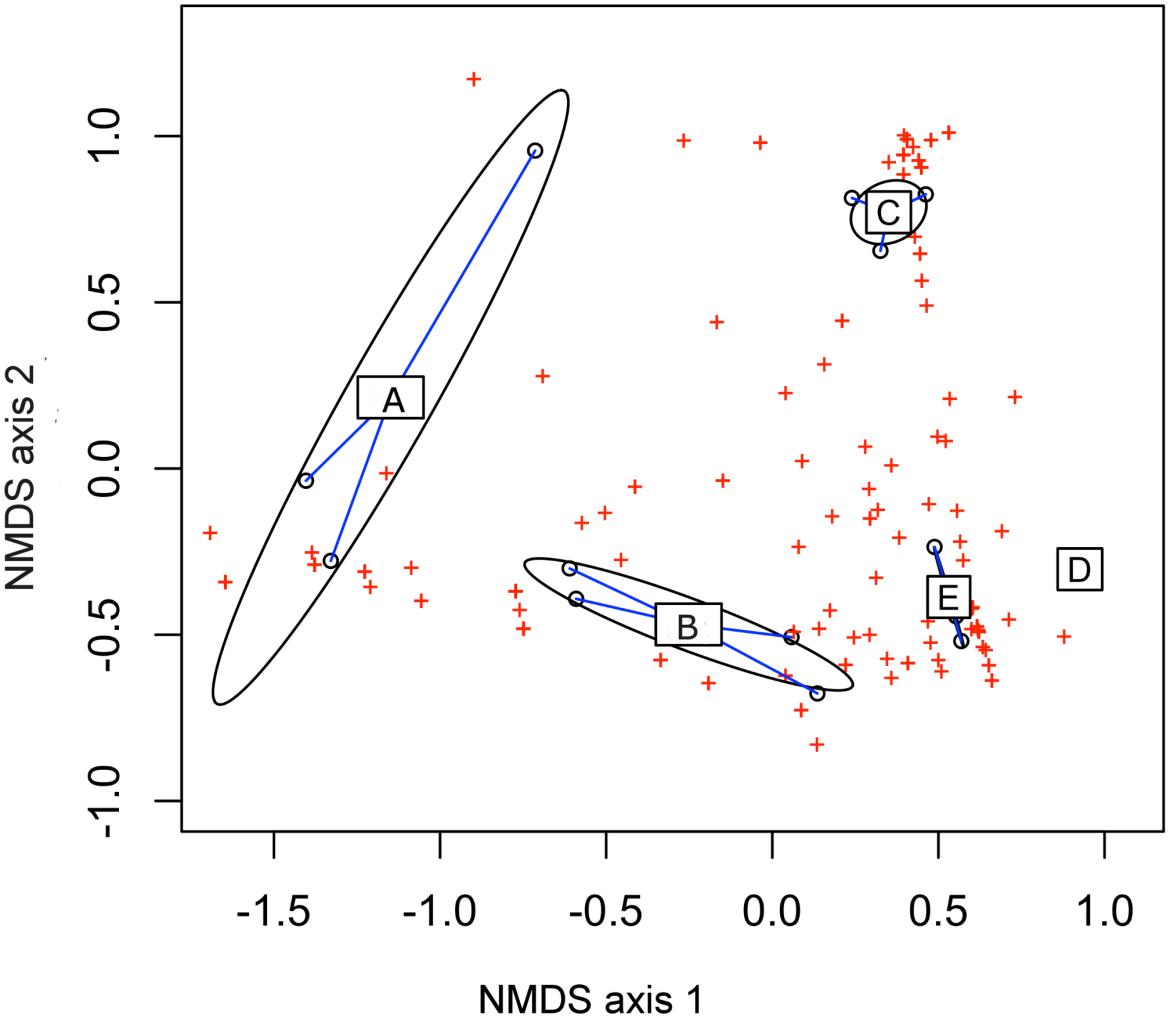
Non-metric multidimensional scaling analysis. NMDS of the fungal community structure of the five groups using the Bray-Curtis distance. Open black circles indicate the single organism while red crosses represent the identified OTUs. Blue lines connect the individual mycobiotas to the centroid values of each group.

Table in S1 Table reports the values of the estimated diversity indices (i.e. Shannon *H* diversity, Pielou's *J* evenness indices and Chao-1) for the fungal community associated with BSF larvae tested in this study. Mycobiota associated with group E had the highest species diversity with an average number (±SD) of 54.3±11.5 detected OTUs, while the lowest value is related to group A (19±6.1). The highest average values of the Shannon and Pielou’s evenness indices were recovered in the community associated with groups of larvae B and C (2.46 and 2.45; 0.67 and 0.62, respectively), whereas group A had the lowest values. The hierarchical clustering showed that the pattern of association of the different fungal communities was congruent with the different analyzed groups; mycobiota associated with each group, characterized by a specific feeding condition, clustered together (S1 Fig). The differences in the fungal communities associated with all five groups of insects were confirmed by an ANOSIM analysis (P<0.001). NMDS analysis shows that the diet can explain the dissimilarity among the BSF-associated fungal communities (NMDS_Bray-Curtis_ R^2^ = 0.87; P = 0.001); two groups of fungi were identified within the BSF fungal microbiota (Fig 3): the first group is associated with larvae fed on chicken feed (A, C), while the second with specimens reared on the two substrates (B, E). Group D, larvae fed on the chicken feed only, but for a longer time, is closer to groups B and E. Venn diagram (S2 Fig) shows that two OTUs are exclusive to group A, 10 to group B, five to group C, two to group D and five to group E. Interestingly, no OTUs in common to all groups were detected.

### Mycobiota composition

**Fig 4:**
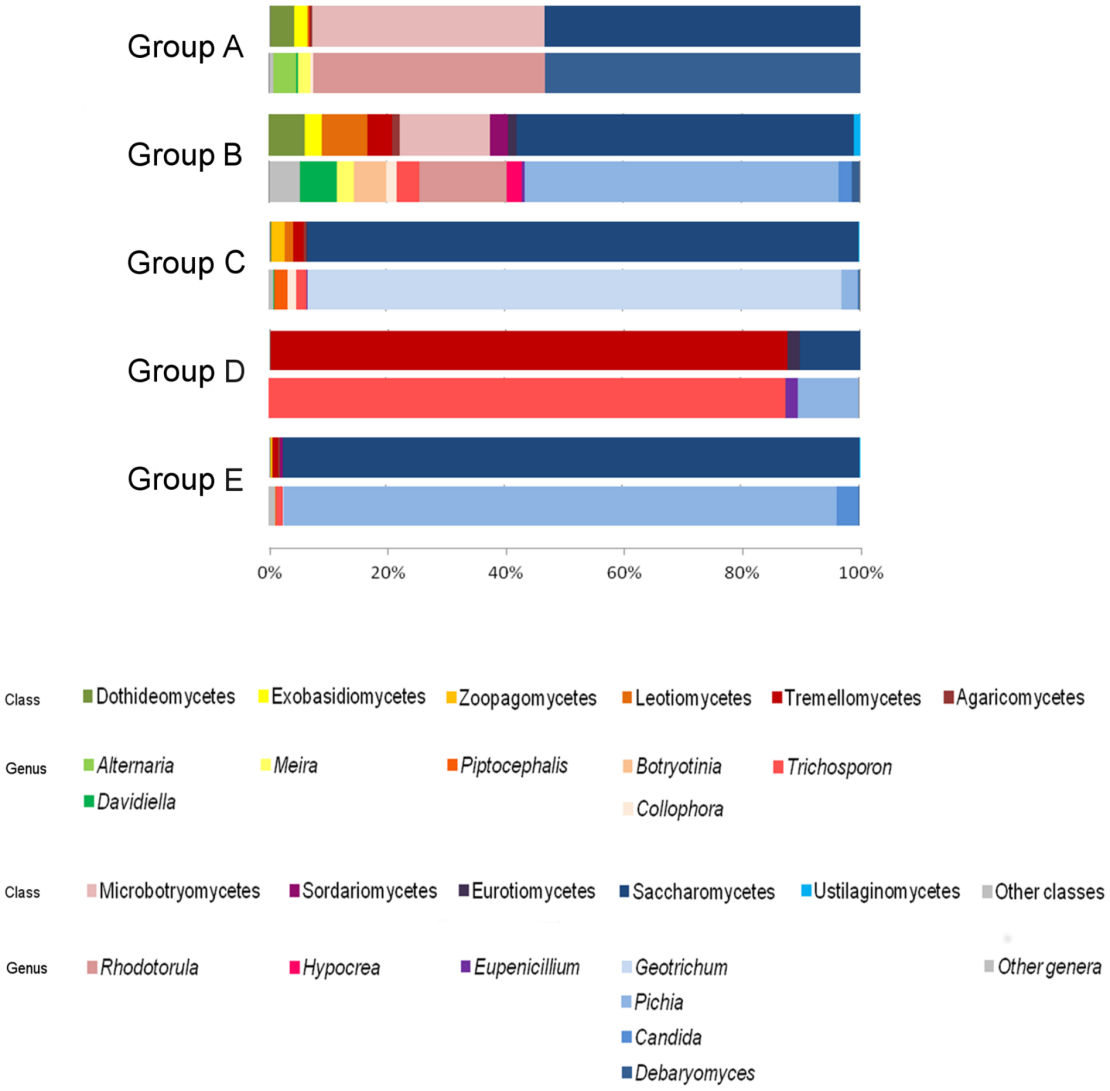
Taxonomic composition of the mycobiota of BSF larvae. Histogram represents the fungal diversity at class and genus level (upper and lower bars, respectively). The average abundance of each group is shown; only the fungal genera/classes with an average abundance >1% are reported, those with an abundance lower than 1% are classified as other classes/genera.

The results of the taxonomic assignment analysis at the phylum and genus levels are reported in Fig 4. The analysis revealed that Saccharomycetes is the most abundant class in groups A, B, C, E (53.4%, 56.9%, 93.4%, 97.7%, respectively), whereas the fungal microbiota associated with group D is dominated by the class belonging to Tremellomycetes (87.7%). In addition to Saccharomycetes, other minor classes were detected in group B: Microbotryomycetes (15.2%), Leotiomycetes (7.7%), Dothideomycetes (6.1%) and several classes with a low abundance (below 5%); among all groups examined, group B appears to have the greatest classes diversity. The second major component in group A is represented by Microbotryomycetes (39.2%). The analysis shows that *Pichia* is the dominant genus in groups B and E (53.2% and 93.7%, respectively), whereas groups C and D are represented almost entirely by the genera *Geotrichum* and *Trichosporon* (90.3% and 87.7%, respectively). Finally, group A is mostly characterized by the genera *Debaryomyces* and *Rhodotorula* (53.3% and 39.2%, respectively).

## Discussion

Insects for feed are processed with their intestinal content, which can harbour different species of transmissible microorganisms. In addition, the microbiota present on the exoskeleton of the insects can be introduced during farming and processing [9]. Accordingly, the safety of insects used as feed or food is a key aspect. In turn, the safety of the insect diet is crucial to ensure the safety of insects as a protein source.

In the present study, the mycobiota of five groups of *H*. *illucens* larvae, characterized by different feeding conditions, has been investigated by culture-dependent approaches (microbiological analysis and molecular identification) and by a culture-independent method (NGS pyrosequencing of the fungal ITS region).

The microbiological analysis shows that group B, larvae fed on chicken feed and vegetable waste (17+4 days) is associated with the greatest species diversity, respect to groups A, C and D, larvae fed on chicken feed only. Vegetable waste promotes the growth of a greater number of fungal species, despite the substrate was heat-treated at 100°C, through cooking. *Pichia*, *Kazachstania* and *Kluyveromyces* genera were isolated only in B and E groups, respect to *Candida* and *Geotrichum* genera, which were associated with the groups of larvae fed on chicken feed only (A, C, D), suggesting that the type of substrate selects a different fungal community. Finally, no ubiquitous yeast species, presents in all groups, was isolated in culture.

By the use of the amplicon-based next-generation sequencing (NGS) technique, a better knowledge of the fungal community of BSF larvae was obtained. The key role of the diet on the mycobiota has been confirmed, in fact no OTUs in common to all groups of larvae were detected (S2 Fig). A further element, proving the impact of the diet on the fungal community, comes from the NMDS analysis (Fig 3). The analysis has shown more similarities between the groups of samples characterized by the same diet: groups A-C, larvae fed on chicken feed and groups B-E, larvae fed on chicken feed and vegetable waste. Interestingly, group D, larvae fed on chicken feed for 21 days, is closer to the groups of larvae fed on chicken feed and vegetable waste. Probably, the substrate at 21 days is more contaminated and the fungal community of group D shows more similarities with groups B and E respect to groups A and C. The results obtained with the microbiological and molecular approaches have been confirmed: group B has the greatest diversity in the fungal community at the genus level, respect to the groups of larvae fed on the chicken feed only. Group E, fed on the same substrates of group B, but for a longer time, was characterized by a less diversified mycobiota, whose dominant genus was *Pichia* (Fig 4). This difference can be due to the fact that some yeasts are able to inhibit the growth of others, because of antagonistic mechanisms, like the production of antimicrobial compounds or toxins, active against fungi and bacteria [46]. The killer toxin production is a well-known antagonistic yeast activity and it has been demonstrated among many yeast genera like *Saccharomyces*, *Candida*, *Cryptococcus* and *Pichia* [47]. According to literature, the majority of the yeasts isolated in this study belongs to species that are regarded as producers of antimicrobial compounds. In order to verify the antagonistic activity of the isolated yeasts against sensible strains, we selected three strains for each yeast species and we investigated their potential antimicrobial activity. All the strains from the species *T*. *asahii* demonstrated an inhibitory activity against the sensible yeasts *C*. *glabrata* and *C*. *lusitaniae*. *Trichosporon* spp. is a basidiomycetous yeast widely distributed in nature, detected in different substrates like soil, food and animal gut [48]. *Trichosporon* is also a medically important genus whose members are able to colonize the gastrointestinal system, respiratory tract, skin, and reproductive system of humans [49]. In particular, *T*. *asahii* (the species we isolated) is reported to be an opportunistic yeast pathogen, part of the human cutaneous microbiota [49]. In our analyses the fungus *T*. *asahii* has been detected in A, B, C and D groups, highlighting that further investigations are required to characterize this opportunistic yeast and its presence in insect-based feed or food.

Regarding the general activity of the yeasts producing killer toxins, the temperature range needs to be taken in account; the optimal condition varies depending on the toxins and the yeast species, but they are generally active and able to produce toxins at temperatures below 30°C [47]. As shown in Hodgson *et al*. [50], generally, the toxin stability decreases with increasing temperature and at 50°C the yeast killer toxins lose their activity. An exception is represented by the toxin produced by the yeast strain *Hansenula mrakii*, which is active at 100°C [51], but considering the high temperature to which the insects for feed can be treated (e.g by meal drying, pelleting and extrusion processes), the toxins produced by yeasts should be eliminated. This is not the case of mold toxins: mycotoxins are secondary metabolites produced by filamentous fungi [14] and are characterized by the property to be resistant to high temperatures; consequently, no treatment appears to be effective [52]. Despite the efforts to control fungal contamination, extensive mycotoxin contamination has been reported to occur in feed and food [53]. In this study, mold isolates were identified as *G*. *candidum*, which is not regarded as mycotoxin producer [44]. Furthermore, the thermal treatments of insects (post-harvest) as well as the choice of the substrate have a great impact on the occurrence and levels of biological contaminants. As reported in different studies, the type of thermal treatment is fundamental, because not all are effective for a complete inactivation of microorganisms and their toxins [54]. The microbiological analysis on fried/boiled insects shows a decrease in microbial load compared to fresh insects, suggesting the importance of a thermal treatment [55]. In this study, we dealt with the fungal community only, but obviously, bacteria and spores resistant to high temperatures should be considered in the selection of treatment; as reported in Klunder *et al*. [56] a heating step of 10 minutes is not sufficient to eliminate bacterial endospores, which remain a potential risk.

A further element to control microbial load could be the use of antibiotics added to the substrate of reared insects. Genta and colleagues [57] investigated at the microbiological level the gut content of *Tenebrio molitor* larvae, reared axenically or adding antibiotics to the substrate: no bacteria and fungi were detected both by adding antibiotics and by rearing the larvae in axenic conditions. In the present study, the substrates provided to the larvae contained a broad-spectrum antimicrobial agent/preservative agent, which has been mixed with the substrate at the beginning of the experiment, and implies only a partial inhibition of the fungal growth; on the other hand, a huge quantity of antibiotics in the substrate could have a negative impact on the insect growth, like larval mass loss and the premature pupation of the larvae [57].

Therefore, even though further researches are needed in order to test the main factors affecting intestinal mycobiota in farmed insect species, present data indicate that: i) the quali-quantitative presence of some genera is strictly dependent on the diet provided to the growing insect; ii) dietary/substrate exposure time has also an influence in defining biodiversity of mycobiota which is probably both transient and environment-dependent. Combining these results, it can be concluded that the substrate/diet on which larvae of BSF have been maintained significantly contributes to the shaping of the structure and taxonomy of the associated fungal communities, even though a time effect cannot be excluded.

## Supporting information

S1 Fig Heatmap cluster analysis. The relative abundance of OTUs, determinated at 97% of identity, is reported in the figure. Coloured scale represents OTUs abundance for each sample.
(TIFF)

S2 Fig Operational taxonomic units shared by the five groups of larvae. Venn diagram shows the exclusive OTUs and those in common to each group (at 97% similarity).
(TIFF)

S1 Table Diversity indices estimated for the fungal communities associated with the analyzed samples. a. Number of OTUs observed in the microbiota of each sample. b. Number of OTUs estimated to be present in the microbiota of each sample. CF: chicken feed; VW: vegetable waste.
(DOC)
