# Supplementary figures and images for: A survey of the mycobiota associated with larvae of the black soldier fly (Hermetia illucens) reared for feed production
Source: PLoS One. 2017 Aug 3;12(8):e0182533. doi: 10.1371/journal.pone.0182533 (PMC5542616; doi:10.1371/journal.pone.0182533)

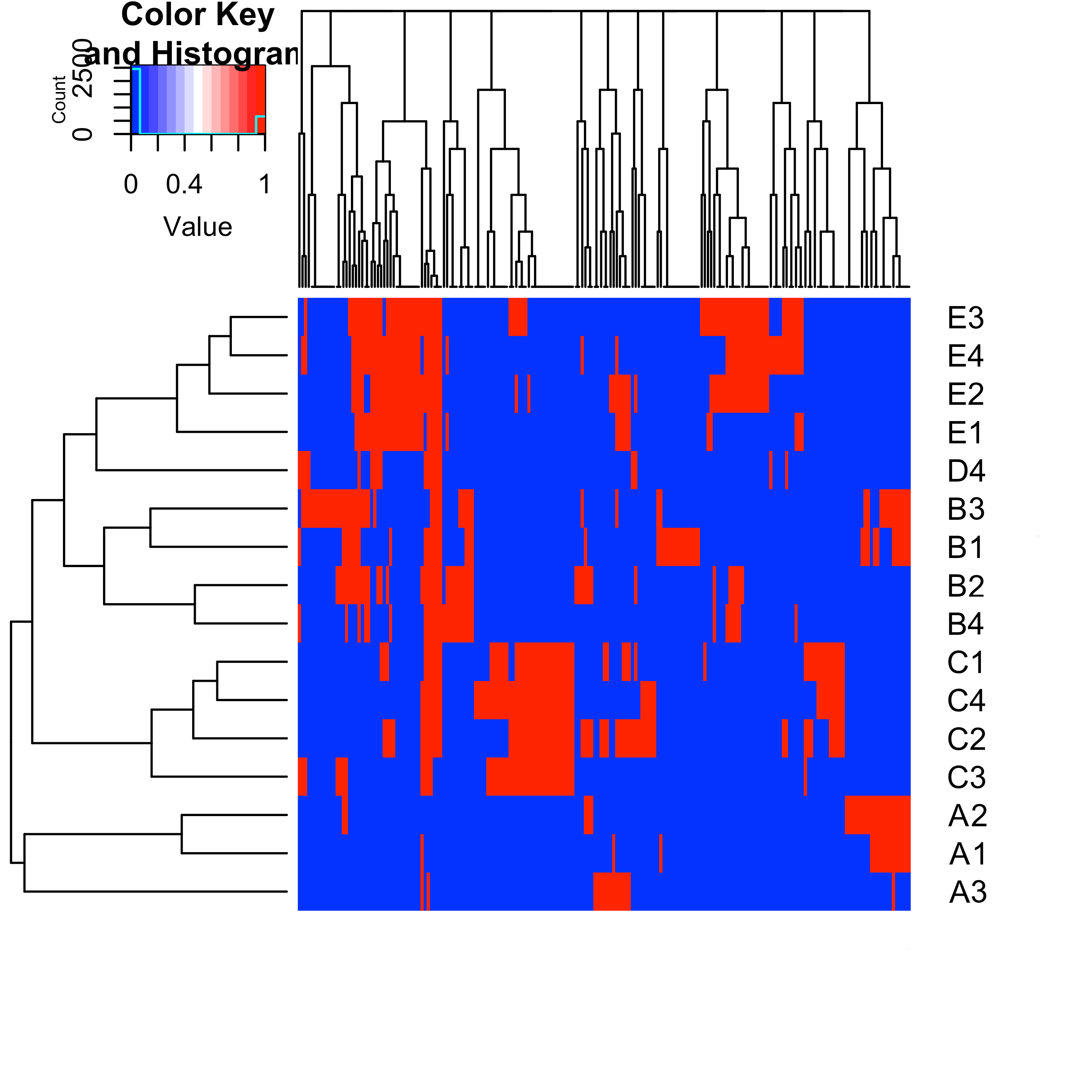

Supplement: S1 Fig — The relative abundance of OTUs, determinated at 97% of identity, is reported in the figure. Coloured scale represents OTUs abundance for each sample. (TIFF) [file pone.0182533.s001.tiff]

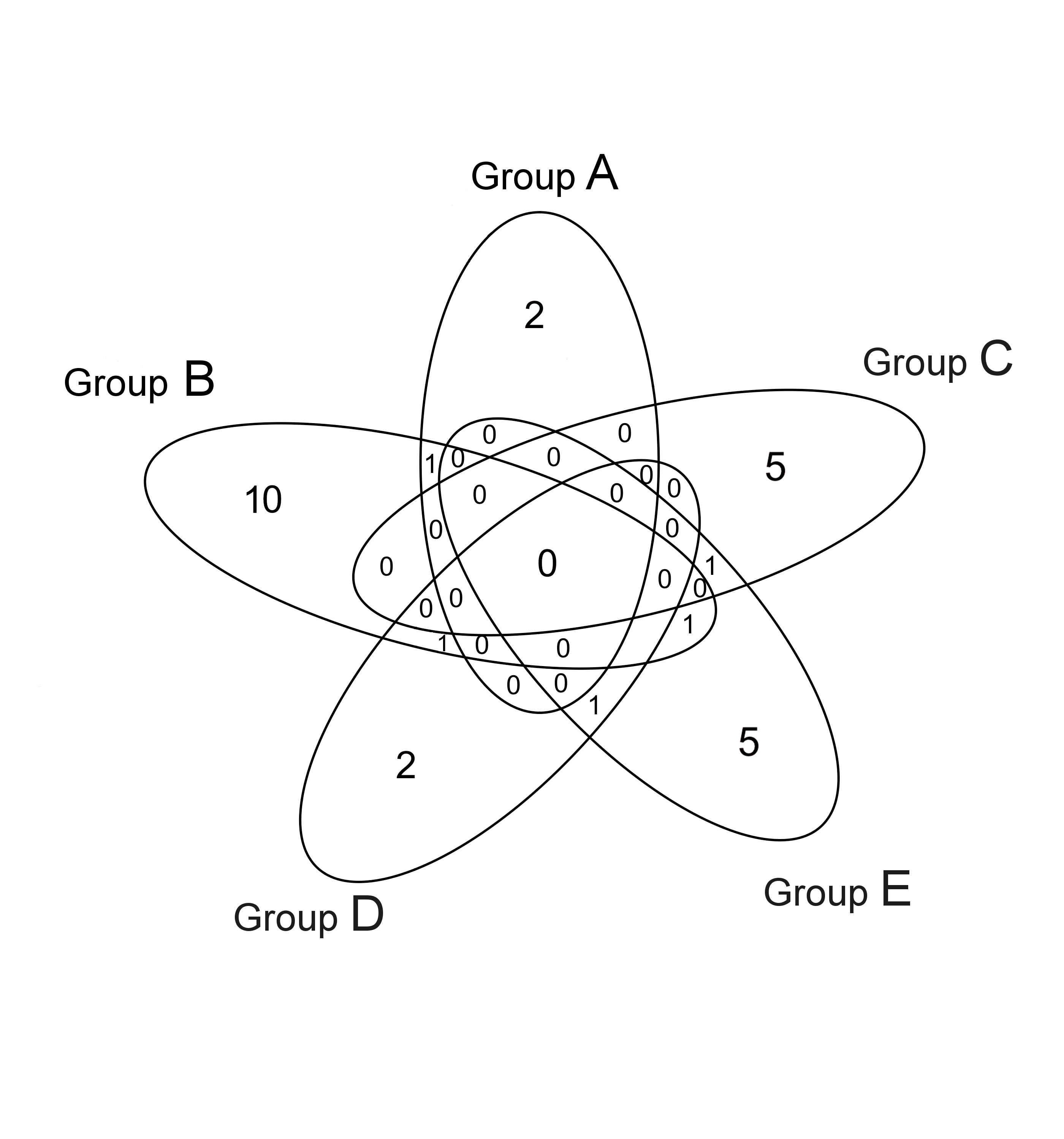

Supplement: S2 Fig — Venn diagram shows the exclusive OTUs and those in common to each group (at 97% similarity). (TIFF) [file pone.0182533.s002.tiff]
