# Supplementary material for: A survey of the mycobiota associated with larvae of the black soldier fly (Hermetia illucens) reared for feed production
Source: PLoS One. 2017 Aug 3;12(8):e0182533. doi: 10.1371/journal.pone.0182533 (PMC5542616; doi:10.1371/journal.pone.0182533)
Supplement: S1 Table — a. Number of OTUs observed in the microbiota of each sample. b. Number of OTUs estimated to be present in the microbiota of each sample. CF: chicken feed; VW: vegetable waste. (DOC) [file pone.0182533.s003.doc]

| **Sample** | **Feed condition** | **S_observed_^a^** | **S_Chao_^b^** | **Shannon *H'*** | **Pielou *J'*** |
| --- | --- | --- | --- | --- | --- |
| A1 | CF 17days | 16 | 17.50 | 1.11 | 0.40 |
| A2 | CF 17days | 26 | 26 | 1.23 | 0.38 |
| A3 | CF 17days | 15 | 15.75 | 0.30 | 0.11 |
| B1 | CF 17days + VW 4days | 43 | 47 | 2.66 | 0.71 |
| B2 | CF 17days + VW 4days | 43 | 49.88 | 2.41 | 0.64 |
| B3 | CF 17days + VW 4days | 48 | 55.20 | 2.55 | 0.66 |
| B4 | CF 17days + VW 4days | 29 | 36 | 2.20 | 0.65 |
| C1 | CF 14days | 54 | 58.09 | 2.45 | 0.61 |
| C2 | CF 14days | 62 | 90.11 | 2.81 | 0.68 |
| C3 | CF 14days | 40 | 57.50 | 2.64 | 0.71 |
| C4 | CF 14days | 53 | 53.33 | 1.93 | 0.49 |
| E1 | CF 14days + VW 7days | 38 | 49.14 | 1.51 | 0.42 |
| E2 | CF 14days + VW 7days | 56 | 65.75 | 1.59 | 0.40 |
| E3 | CF 14days + VW 7days | 65 | 72.58 | 1.79 | 0.43 |
| E4 | CF 14days + VW 7days | 58 | 71.60 | 1.47 | 0.36 |
| D4 | CF 21days | 19 | 26.50 | 1.36 | 0.46 |
